# ‘When you talk to someone in a bad way or always put her under pressure, it is actually worse than beating her’: Conceptions and experiences of emotional intimate partner violence in Rwanda and South Africa

**DOI:** 10.1371/journal.pone.0225121

**Published:** 2019-11-14

**Authors:** Erin Stern, Andrew Gibbs, Samantha Willan, Kristin Dunkle, Rachel Jewkes

**Affiliations:** 1 Gender Violence and Health Centre, London School of Hygiene and Tropical Medicine, London, United Kingdom; 2 Gender and Health Research Unit, South African Medical Research Council, Pretoria, South Africa; Purdue University, UNITED STATES

## Abstract

Emotional intimate partner violence (IPV) is extremely common and has significant health and social consequences, yet typically receives much less attention in research and programming than physical and sexual IPV. This limits our understanding of how women experience and understand emotional IPV in different settings, which is required to inform effective prevention and response. This paper draws on qualitative data collected in mixed-methods impact evaluations of two IPV prevention programmes conducted as part of the What Works to Prevent Violence Against Women and Girls Global Programme. In doing so, we seek to develop a more nuanced understanding of the forms, causes and consequences of emotional IPV in heterosexual relationships in two distinct African settings. We draw on two rounds of in-depth interviews conducted with 15 women in South Africa and three rounds of interviews conducted with 57 women and men in Rwanda, all of whom were participants in the programmes, around their experiences of and conceptualizations of emotional IPV. Thematic analysis around emotional IPV was conducted and compared across both data sets, informed by a cross comparative analysis approach. The analysis found that the categories or types of acts perceived as emotional IPV by women experienced were similar across settings. Women in both contexts described public humiliation, control of mobility, access to housing and silencing as important categories of actions experienced emotional IPV. These types of emotional IPV were underpinned by similar patterns of gender inequalities, with the intention for men to assert control and power over women. The specific acts through which these categories of emotional IPV manifested in the two settings arose from contextual differences related to the social structure of relationships and dominant social norms. This analysis highlights commonalities in the underlying categorial understanding of IPV in two distinct African settings, and well as the differences in specific manifestations which stem from the social context of relationships. In doing so, we highlight both broad categorical areas of IPV that may be important to address in future research and prevention programming, as well as affirming the need for information on context-specific manifestations of emotional IPV to inform local intervention programmes.

## Introduction

Emotional violence is generally agreed to encompass forms of aggressive behaviours that cause psychological injury, rather than physical or sexual harm. Emotional intimate partner violence (IPV) in heterosexual relationships is commonly perpetrated by men against women in order to achieve control and dominance in relationships, and as a form of hurt or punishment. While men also experience emotional IPV, due to systematic power hierarchies, women are more vulnerable to all the different forms of IPV, including emotional IPV. Quantitative research consistently finds that emotional IPV is more prevalent than physical or sexual IPV [1]. In 12 Demographic and Health Surveillance studies from 10 African countries, lifetime emotional IPV was typically reported as often, or more often, than physical IPV by women [2]. In a four-country population-based study from Asia and the Pacific, lifetime emotional IPV was the most common form of IPV women reported experiencing, ranging from 30.0% in Sri Lanka, 38.3% in China to 64.9% in Cambodia, and 69.1% in Papua New Guinea, while lifetime physical and/or sexual IPV was 27.8%, 38.7%, 25.3% and 67.5% respectively [3].

Emerging research suggests that emotional IPV also has significant health impacts independent of those arising physical and sexual IPV. A range of studies, including longitudinal studies, have found that women experiencing emotional IPV are more likely to experience adverse mental health outcomes, including depression and suicidal ideation [4, 5]. An analysis of data from young women (18–30) in South Africa [4] found that a range of potential measures of emotional IPV showed a consistent positive association with higher depression scores and suicidal ideation. Other studies have shown that women’s experience of emotional IPV is associated with psychosomatic conditions (such as nervous twitching, cramps and paralysis, and dizziness) [6] as well as fear, anxiety, reduced social support, and compromised self-esteem [7].

Despite the growing evidence of both the high prevalence of emotional IPV and its independent impact on women’s health, beyond physical and sexual IPV, there remain concerns about how to best conceptualize and understand it. One major concern is the extent to which emotional IPV is contextually dependent versus generalizable across settings. In comparison to physical and sexual IPV, where there are specific and relatively standardized behavioural measures that perform well across different settings [8], emotional IPV is less clearly defined and more difficult to identify and operationalize. This is visible in part through the proliferation of diverse terms for emotional IPV, including psychological abuse/harm, verbal abuse, as well as emotional abuse/harm. There is also a lack of clarity about whether emotional IPV can be usefully differentiated from other types of male behaviour that seek to control women. For instance, emotional IPV can include what has been termed ‘controlling behaviours,’ such as unfounded jealousy, restricting movements, dominating decisions and expecting obedience, and controlling clothing choices [7]. As such, the manifestations of emotional IPV that have been considered in work on this topic are varied and include personal insults, belittling a partner in front of others, isolating a partner’s friends and family, damage to property, deliberately flaunting another sexual partner, intimidation or threats (e.g. by yelling or breaking things), threats to end the relationship, threats to harm him/herself, threats against the partner, and threats of violence against or actual harm to loved ones, pets, or property [1, 4]. There remains very little cross-setting comparative analysis of how participants in heterosexual relationships experience and understand emotional IPV and how this may be grounded in context-specific gender inequalities, and/or understandings of heterosexual relationships.

To address this question, we draw on in-depth interview data collected as part of mixed-methods impact evaluations of two IPV prevention programmes conducted as part of the What Works to Prevent Violence Against Women and Girls Global Programme (www.whatworks.co.za): the Stepping Stones and Creating Futures study in South Africa and the Indashyikirwa (“Agents of Change”) study in Rwanda. Although these programmes and their accompanying impact evaluations were quite different, the overlap in approaches to analysis and close cooperation between investigator teams offers a unique opportunity for cross-contextual secondary data analysis on this important topic. A cross comparative analysis was applied to reflect on common categories of acts women perceive as emotional IPV, which is valuable for better conceptualizing and operationalizing the often rather indistinct notion of emotional IPV. This comparative analysis deepens our understanding of the categories, causes and consequences of emotional IPV experienced by women in two Sub-Saharan African settings, the focus of this paper. It also points to the ways in which prevention programmes and measures used in evaluations should consider how emotional IPV is experienced in diverse settings and in different social contexts of intimate relationships. Indeed, the strength of this paper lies in identifying common domains across divergent studies and settings, as well as contextual variations in manifestations of emotional IPV.

## Settings and programmes

### South Africa

South Africa has, over the last ten years, seen a significant development of policies and national programmes focused on preventing and responding to IPV. A review of the 2007–2011 National Strategic Plan for HIV/AIDS and STIs noted that it included a significant focus on the association between IPV and HIV-acquisition and included objectives around both preventing and responding to IPV [9]. More recently, there has been a push to prevent IPV amongst young women and girls, through the ‘She Conquers’ campaign and the DREAMS initiative (funded by the USA government).

Despite a strong policy framework and a level of commitment by government and civil society to preventing IPV, IPV remains incredibly common, particularly in urban informal settlements. Although no nationally representative data exist, baseline data from the Stepping Stones and Creating Futures (SSCF) trial with young women (18–30) in urban informal settlements (discussed further below) found that 59.6% reported past-year experience of physical IPV and 29.4% sexual IPV [10]. This compares to population-based data from the Gauteng Province where 13.2%, report past year physical IPV 7.8% sexual IPV [11]. Emotional IPV was also incredibly common in the SSCF data, with 78.1% of young women in informal settlements reporting past-year experience of this [10]. The most common form of emotional IPV reported was being insulted (64.7%) while over a third (38.8%) reported being humiliated in front of others and 40.7% reported being threatened by their partner in the past year.

In South Africa, there has been a continual decline in marriage rates as a result of the changing political economy, particularly the apartheid system and subsequent economic restructuring that occurred in the 1990s [12]. Overall, in 2001, only 30% of South Africans were formally married, although cohabitation remained much more common [12]. Residents of urban informal settlements are younger on average than those in formal settlements, as well as significantly poorer and less likely to be employed. Crime in these areas is high and services and infrastructure are notably lacking [13]. In these contexts, sexual and romantic relationships are often complicated and fluid, with multiple and concurrent sexual partners, including transactional relationships, common to the point of being almost normative [12].

### The Stepping Stones and Creating Futures intervention

The Stepping Stones and Creating Futures intervention was a group-based participatory intervention that sought to prevent IPV through strengthening livelihoods and transforming gender relationships amongst young (18–30) women and men living in urban informal settlements. Participants voluntarily self-selected into the programme based on eligibility criteria, which included being unemployed, resident in a programme informal settlement, aged between 18 and 30, and not in school. The intervention was implemented by trained peer facilitators from Project Empower, an NGO based in eThekwini Municipality, the third largest urban area in South Africa, and on the eastern seaboard of the country. Peer facilitators ran single sex groups comprising of approximately 20 participants each. Sessions covered topics including HIV-transmission and prevention, gender roles, communication, livelihood strategies, and savings and were based on participatory techniques [14]. The young women in the SSCF intervention were all resident in urban informal settlements around eThekwini. Baseline quantitative interview data suggested that they experienced significant levels of childhood trauma, poverty, and on-going violence—including IPV—in everyday life. Analyses of these data showed that women’s experiences of physical and/or sexual IPV were associated with higher levels of food-insecurity, male controlling behaviours, quarrelling with partners, and more alcohol use [10]. The majority of these young women did not live with their male partners, rather they lived either on their own or with friends or family members. Very few worked on a regular basis, surviving on financial support from male partners, extended family, their children’s biological fathers, child support grants, and a range of other strategies including transactional sex. Male partners were assumed to have additional sexual partners, and infidelity alone was not normally a reason to end a relationship. Many of the young women also had multiple sexual partners, including both romantic and transactional partners, and relationships tended to be unstable [15].

### Rwanda

In the last few decades, Rwanda has witnessed a significant growth of policies, laws, and programs supporting women’s rights, some of which are intended to better prevent and respond to IPV. These include the 2008 adoption of the Prevention and Punishment of Gender-Based Violence Law, with the minimum penalty for IPV being six months in prison, while sexual abuse or rape, leading to terminal illness or death of one’s spouse, can lead to life imprisonment [16]. At the same time the Rwandan government has supported the development of initiatives to tackle gender-based violence (GBV) and IPV including prevention clubs in schools and universities; GBV prevention committees at the village level; parents’ evening forums to raise awareness, identify, and assist victims of violence; and GBV desks at the Ministry of Defense and National Police [17]. In 2009, the Rwanda National Police and Ministry of Health launched a nationwide rollout of One Stop Centers for women and children who experience violence; these offer free medical care, psychosocial support, and legal services to victims of IPV and child abuse, and emergency accommodation for a few days [18].

Despite the significant policy and programmatic work of the Rwandan government, IPV remains a persistent phenomenon. The 2014/2015 Rwanda Demographic and Health Survey (DHS) found that one third of women (31%) reported a lifetime prevalence of physical IPV, 12% reported any sexual violence, and just over a quarter (27%) reported emotional IPV [19]. When emotional IPV is separated out into individual items, a quarter of women (23.3%) reported ever being insulted or made to feel bad about herself, while 16.7% reported their partner said or did something to humiliate her in front of others. Women also reported high levels of spousal controlling behaviours, including a third (35%) reporting their husbands/partners were jealous or angry if they talked to other men, and that husbands/partners insisted on knowing where they are at all times (29%), and they do not permit them to meet female friends (14%).

In Rwanda, as in many global contexts, formal marriage is a critical pathway to achieve societal status and acknowledged adulthood for men and women [20]. The status benefits of marriage are encouraged by the Rwandan Government, which has taken steps to increase the proportion of couples in formal, civilly registered marriages in conjunction with the growth of national policies, laws and programmes intended to guarantee the rights of legally married women. Of note is the 1999 Law on Matrimonial Regimes, Liberalities and Successions, which established women’s rights to inherit land for the first time in Rwanda [21]. In Rwanda, there is a strong cultural prohibition against sex outside of marriage or at least formalised co-habitation [19]. The 2014/2015 DHS showed a third of women and men of reproductive age are in formal marriages (35%; 33% respectively) and 14% of women and 17% of men are in informal marriages, whereby they live together without having civilly registered their marriages. Several studies have found that some men resist entering intro formal marriages to avoid the accompanying rights for women, especially the right to property, and to preserve the traditional male headed household dynamic [22, 23]. While Article 206 ‘Equality of Spouses’ of the Rwandan Civil Code defined men as the ‘head of the household’, codifying men’s authority in the family [24], this article was amended in October 2016 to recognize both spouses as having the same rights and obligations in the household, with each owing the other mutual fidelity, help and assistance.

### The Indashyikirwa programme

Indashyikirwa “Agents of Change” was a community IPV prevention programme funded by DFID Rwanda and jointly implemented by CARE International in Rwanda, Rwanda Women’s Network (RWN) and Rwanda Men’s Resource Centre (RWAMREC) from August 2014 through August 2018 [25, 26]. The programme ran across fourteen sectors spread among seven Districts in Eastern, Northern and Western provinces of Rwanda, and in predominantly rural, widely spread communities. There were four main components to the programme: (1) Intensive participatory training with couples (Couples’ Curriculum); (2) Community-based activism with a sub-set of trained couples; (3) Direct support to survivors of IPV through the women’s safe spaces; and (4) Training and engagement of opinion leaders. The programme sought to reduce experiences and perpetration of IPV, and shift harmful social norms condoning IPV. The programme also strove to support equitable, non-violent relationships, and ensure more supportive and empowering responses to survivors of IPV. The eligibility criteria for couples to enroll in the curriculum and as activists was that at least one partner (often the female partner) should be a member of CARE Rwanda’s micro-finance village savings and loans association, be between 18–49 years of age, and that the couple had been married or living together for at least 6 months. Both partners of couples that met these criteria volunteered to participate in the curriculum, and were selected via a lottery, given the high demand for this programme.

Although this paper does not compare the impact of the two programmes on addressing emotional IPV, both interventions had efforts to encourage participants to consider emotional abuse and reflect on it in the context of their lives. Such insights were useful for the interviews and the rich data that informed this paper. In both interventions, experiences of IPV was not a criterion for enrolment, yet the quantitative baseline evaluations with participants enrolled in both programmes found very high levels of IPV, indicative of how both programmes were reaching those who could benefit.

## Methods

### Data collection South Africa

In South Africa 15 women were recruited from two intervention clusters to participate in the qualitative study, which are detailed in Table 1. Two interviews at baseline (immediately before and at the beginning the programme) were undertaken with each woman. The first interview was primarily about background and basic socio-demographic information, as a way to establish a rapport between the interviewee and interviewer. The second interview was longer and focused on relationships, children, livelihood strategies and overall life experiences. Each interview lasted 45 minutes to 1.5 hours. Interviews were conducted in the privacy of women’s homes, or in public spaces where audio privacy could be achieved.

**Table 1:** Interviewees South Africa.

Participants	Number Interviewed	Timing	Recruitment Criteria	Scope of Enquiry
Women participating in the Stepping Stones and Creating Futures intervention	15	February-March 2016; October-January 2017	Eligible for the Stepping Stones and Creating Futures trial; 18–30, normally resident in the informal settlements; able to consent to participate. In one of the two selected clusters for fieldwork	Background, livelihood strategies, relationships, children, reproductive decision-making and violence.

One female ethnographer/fieldworker undertook all the interviews, in isiZulu and audio recorded them.

### Data collection Rwanda

Qualitative research as part of the overall impact evaluation was conducted in three intervention sectors (Rurembo Sector, Western Province; Gishari Sector, Eastern Province; and Gacaca Sector, Northern Province), which were purposefully selected to represent a diversity of environments including rural and peri-urban locations. Table 2 details the interviews across different time points that were drawn on for this paper including at baseline (after participants were enrolled in, but before starting, the intervention), midline (6–12 months after involvement with the intervention) and endline (12–24 months after involvement with the intervention).

**Table 2:** Interviewees Rwanda.

**Participants**	**Number Interviewed**	**Timing**	**Recruitment Criteria**	**Scope of Enquiry**
Both partners of couples (separately)	28 = 14 men and 14 women (4–5 men and 4–5 women per province)	November 2015 (after enrolling but before participating in couples curriculum); May 2016 (after completing couples curriculum); May 2017 (one year post midline)	Diversity of informally and formally married couples recruited by RWAMREC staff, for being primary distinction among couples enrolled in curriculum	Couples’ expectations of programme, experiences of conflict and IPV, communication skills and joint decision-making; how involvement with Indashyikirwa continued to impact their relationships including difficulties with changes
Opinion Leaders	11 (3–4 per province)	November 2015 (after enrolling but before participating in opinion leader training); November 2016/ June 2017 for 3 additional opinion leaders added; May 2018	Diversity purposefully recruited by RWN staff including government leaders, members of GBV committees or National Women’s Council and religious leaders	Expectations of programme, experiences around IPV prevention and response; Impressions of Indashyikirwa training and whether involvement in the programme has influenced actions for IPV prevention and response
Women’s Safe Space Facilitators	3 (1 per province)	May 2016 (after completing programme training and beginning role as facilitators); June 2017; June 2018	1 facilitator per safe space recruited by RWN staff	Motivations to be facilitators and their impressions of the programme training; Perceived impact of the safe spaces and the support they receive as facilitators
Community Activists	12 = 6 men and 6 women (2 men and 2 women per province)	November 2016 (after completing activism training and beginning activist activities); May 2018	Activists who had not participated in couples interviews recruited by RWAMREC staff	Impressions of the activism training, what motivated them to continue as activists, what they had been doing recently as activists, and whether they faced any challenges

The interviews lasted approximately 1–1.5 hours and were conducted at locations deemed appropriate and private by participants but ensuring audio-privacy. Two female Rwandan qualitative researchers external to the programme conducted the interviews with women’s safe space facilitators, attendees, opinion leaders, community activists and female partners of couples. Two male Rwandan qualitative researchers conducted the interviews with opinion leaders and male partners of couples. All of these interviews were conducted in Kinyarwanda and audio recorded. All field staff had significant prior experience and participated in rigorous GBV-related interview training facilitated by the first author.

In both studies, emotional IPV was not asked about directly in interviews but came up when asking participants about relationship quality, overall experiences of violence, and in the case of the data from Rwanda, impacts of the programme. In South Africa, we did not enquire about programme impacts, as all interviews with women that informed this analysis were conducted before the programme was significantly underway, whereas women’s interviews from Rwanda were conducted at baseline, midline and endline throughout the programme implementation.

### Ethical considerations

Ethical approval for the Stepping Stones Creating Futures study was received from the South African Medical Research Council (EC006-2-2015) and the University of KwaZulu-Natal (BFC043/15). Participants signed informed consent forms for participation in the main trial, and separately for involvement in the qualitative study. No specific reimbursements were provided to participants for qualitative interviews, but the fieldworker would buy some basic food and drink to share with the participant.

In Rwanda, ethical approval was obtained from the Rwandan National Ethics Committee (RNEC) (REF: 340/RNEC/2015). Secondary approval was also obtained from the South Africa Medical Research Council (REF: EC033-10/2015) and the London School of Hygiene and Tropical Medicine. Before each interview, participants were given a chance to ask questions and to provide written or oral informed consent. All participants were given 2000 Rwandan Francs (approximately USD 2.50) as a token of appreciation for their participation.

### Data analysis and interpretation

All interviews were audio recorded, transcribed and translated verbatim into English. The transcripts were then analysed by the first author (Rwanda) and second author (South Africa) using a thematic coding framework via NVivo 11 (QSR International, Victoria, Australia) for the former and Microsoft Word for the latter. For the data from South Africa, the third author had many in-depth discussions with the interviewer, who was involved with the analysis. They discussed the context of the interviews and the interview observation notes collected by the interviewer. For the data from Rwanda, the first author debriefed with the qualitative researchers after data collection to capture their initial impressions, non-verbal and con- textual insights. These research summaries were used to inform the analysis.

For both studies, thematic analysis was conducted to uncover predominant themes and provide a rich, detailed and holistic account of the data [27]. For this paper, we used focused coding around emotional IPV and controlling behaviours as they emerged from the data. We identified areas that had reported significant consequences for women but were not forms of physical or sexual IPV. In writing this paper, the first and second authors shared coded data around emotional IPV for the respective data sets and carried out joint interpretation and comparative analysis. A comparative case study approach guided the analysis and presentation of the findings. This approach justifies the importance of examining processes of sensemaking as they develop over time, in distinct settings, with regards to a similar phenomenon [28]. This approach cautions against essentializing notions of culture or nationality and emphasizes the importance of comparing across communities and states, which in doing so, prioritizes a more nuanced consideration of context.

The first author regularly workshopped the emerging findings from Rwanda with the Indashyikirwa senior programme staff to allow for their insights to the interpretation of the data and to validate contextual insights. The second and third authors regularly discussed the findings with the programme staff in South Africa. Indeed, both studies took multiple steps to ensure quality of the data including by conducting multiple interviews with the same beneficiaries, ensuring thick descriptions by validating the insights with interviewees and programme staff, and conducting detailed, thematic analysis.

Given the sensitivities of the data, and exploratory nature of this study, no demographic identifications are used. All participants’ names have been changed to pseudonyms.

## Findings

For this paper, we focus on emic, emerging, categories experiences of abuse that women spoke of having experienced from their male partners, which we have conceptualized as emotional IPV. These categories of emotional IPV were common across both settings, as indicated by the thematic analysis, yet the specific manifestations were often quite different depending on context.

### Humiliation

#### Forms of humiliation

Being humiliated publicly and privately was one of the most common forms of emotional IPV described by participants in Rwanda and South Africa. In Rwanda, this form of humiliation included being undermined by being called ‘stupid’, ‘useless’, ‘idiot’, and also considered sub-human by being labelled a ‘dog’ or ‘property’. It also included statements that undermined one’s value as a spouse. For instance, Josephine, a female partner of a couple that complete the Indashyikirwa curriculum, said at midline: “He was always insulting me that I am useless to him and he was like, you, you are not even a wife! You are just the source of our problems.” Verbal humiliation could include wives being negatively compared to other women, which was said to have both emotional and physical consequences linked to this source of anxiety. Annette, a women’s safe space facilitator explained:

Emotional violence is for example when your husband compares you with other women saying for example: ‘that woman is better than you. I should not have married you!’ that is emotional violence. By that, he hurts you and it affects your body too.

Several women in Rwanda reported shameful public experiences of verbal abuse and humiliation by their spouse. Carene, a female partner of a couple that completed the Indashyikirwa curriculum, shared at midline:

He would go on the hill and call me loudly even with other people listening to him and he would be like: ‘you stupid, where are you at this time?’ or when we were with other people he would say in public: ‘do you think you are a woman?’

In South Africa, young women in urban informal settlements similarly described being humiliated by their partners, and commonly framed this around being ‘made fools of.’ The South Africa women interviewed used the same framing as women in interviews in Rwanda, but largely ‘being made a fool of’ here was about their boyfriend cheating on them in public ways:

Sbenzile: Especially when you now live at his home, there is that thing. He starts seeing other women outside, there in the area. And you start feeling like a fool. You are the one that wakes up early in the morning, heat up the water, you do things for them…You see it’s exhausting.

Women in South Africa also described being humiliated and upset by arguments that became public knowledge. While the hurtful comments and abuse that were involved in these arguments upset them, they perceived that it was as they became more public that it became most distressing:

Thembeka: I don’t talk much when he does such things, I just keep quiet and watch him. He would come back and shout/ swear at me… He would fight me with me, shouting causing a scandal in the community.

Similarly, for Olwethu, while arguments and disagreements in a relationship were common and something that could be worked through, they became humiliating when they were made public, and discussed in public settings:

Olwethu: If you have problem with your partner it’s important to sit down and talk- not to shout and hit them. Okay maybe you can shout at them but it needs to be in the confine of your own home, it’s not okay for them to go around talking about the fight.

#### Consequences of humiliation

Many participants in Rwanda identified negative consequences of verbal abuse and humiliation, including extreme sadness and anxiety. Theophile, a male partner of a couple, shared his perception of the severity of verbal abuse at midline: “Even when you talk to someone in a bad way or you always put her under pressure, it is actually worse than beating her.” A few Rwandan opinion leaders and several partners of couples and community activists lamented the extent of men’s regular verbal abuse against their wives, given the negative emotional and relationship consequences. At baseline, a local government leader emphasized:

Claude: We have at least four households out of ten, where people always tell words that hurt; you cannot find signs that they have been beaten or injured but you would find that the man is not proud of his wife or the wife is not proud of her husband.

At endline, one government leader in Rwanda suggested that men’s use of emotional IPV to cause humiliation or embarrassment may be increasing as he perceived physical IPV to be reducing:

Theophile: There are no more men who still beat their wives, they instead try to make them uncomfortable or embarrassed. They now commit violence in a different way because they know that if you beat your wife you will be imprisoned.

A women’s space facilitator in Rwanda lamented how women were less likely to report or seek help for experiencing verbal abuse, as compared to physical and sexual IPV, despite the significant consequences and commonality:

Agnes: There is a couple I helped, when the man arrives home and he finds that his wife is cooking food, he starts telling her: ‘so you think that you have cooked? What did you really cook? Madam X is the one who really knows cooking!’ but then next morning, that woman holds on and doesn’t say anything to anyone about this! I went to see that woman and told her: ‘why can’t you just say it so that it can get out of you? She was like: ‘what would it solve? I just suffer inside.

In South Africa, many women conveyed a strong sense of inevitability that their partners would be unfaithful, which could be tolerated and was not necessarily directly humiliating, as long as it was done in secret. If men successfully hid their casual or side partners, this was considered a demonstration of love as the primary relationship with the girlfriend remained unchallenged, but public cheating was considered shameful and upsetting: “No it is not right to have many girlfriends, it is best for someone to hide it at least, it’s not okay for you to see that.” (Nkanyezi) For instance, Thembeka described the negative impact of her boyfriend openly cheating on her while she was pregnant with their first child:

But our relationship was not the same because, I now had a child. And you know men when you are having a child, at 7 months you have to stop having sex, whilst you are doing that, he is busying with other women, asking them out, you know these things. All these things that make you unhappy, you hear things, like he is in a relationship with so and so.

### Jealousy and controlling behaviours

Another common form of emotional IPV described across both settings was men’s effort to control women’s movements and association, underpinned by men’s suspicions that their spouse/girlfriend may cheat on them. Common forms of control of movement in Rwanda included prohibiting women from visiting family members and friends, attending church, attending social events, or participating in government community meetings. Several participants in Rwanda identified husbands/partners’ jealousy and control of movement as a form of violence and denying women’s freedom. At the endline interview, one female community activist noted how her husband prevented her from attending community meetings and the double standards compared with her husband because she is a woman:

Sidonie: When I told him I am going to attend the monthly community work, he said, no, you won’t go there. I am the one who will go there. When he was coming from there and I asked him, can you at least share with me the conclusion taken from the community work meeting, he refused to tell me. When I went somewhere and I told him after coming home, he would be sad and ask me why I didn’t tell him in advance. I asked him, why are you sad? Why did you also go somewhere the other time without telling me? Did I take it badly? And he would be like, ‘it is because you are a woman.’

Men’s jealousy in Rwanda could be triggered by women bringing home income or possessions, under an assumption that there came from another man and this was linked to the dominant social norm of men as financial providers. A few women noted how this jealousy led to their spouses destroying or taking away possessions they brought home, including phones or clothes; a form of economic abuse. Control of women’s movement, hindering their ability to socialize, and punishing perceived transgressions, (e.g. through destruction of property) could generate solitude, anxiety and sadness. As Immaculee, a female partner of a couple expressed at endline:

I could not attend any meeting because he had told me: ‘you should not attend any meeting. You must stay here.’ “When I had bought some clothes he would ask me: ‘who did you get these clothes from?’ he would burn some of them, and took away others. He was saying that other men give me money and that he doesn’t want me to have many clothes so others don’t flirt with me. Because I was in solitude, I wouldn’t take any decision. I was always afraid and sad.

Similarly, in South Africa, women described multiple ways in which their boyfriends sought to isolate them from others, primarily under the guise of being jealous and/or having ongoing suspicions that their girlfriend was cheating on them. There were three main patterns of this amongst young South African women: policing of cellphones, constantly checking where women were, and limiting who women could go out with. The majority of South African respondents reported that their boyfriends carefully policed their (women’s) cellphone use as men were concerned that women were going to cheat on them and this would be facilitated through cellphones. This included men checking who phoned them and reading text and WhatsApp messages and this leading to arguments. Khanyisile described how answering calls could lead to arguments: “when the phone rings and I go outside and answer it, and then he asks me why I doing what I am doing up until he sees that it’s someone who is asking me out, then we end up fighting.” As WhatsApp had become more popular, boyfriends often tried to check women’s messages on WhatsApp. In the case of Langa, she described how her boyfriend tried to stop her entirely using the messaging system.

Some women reported continually being checked up on by their boyfriends, which focused on control of their sexuality through control of their mobility. One woman described having to continually report where she was:

Sthelo: My ex-boyfriend would choose the people he wanted me to hang out with. I would tell him that I am going to church and he would ask me who I was going with, If I told him I was going with friends, he would refuse and only agree if I was out with my family. He would tell me when and where I go drinking. I would have to report everything to him even when I went to work, he would walk me to the bus stop, we would wait for the taxi, he would watch me get in the taxi and I had to call and tell him all the details… ‘I got of the taxi… I am at spar buying lunch… I am walking to work… I arrived at work…I am about to start working… I am on lunch… I am finished working… I am in a taxi making my way back.’

Other women reported that their boyfriends tried to limit who they saw, and what they did to specifically prevent them from spending time with other men:

Nondo: I wanted to go to a party. I told him [my boyfriend] I was invited to a party and he said ‘you are not going’, and I said, but you don’t even know who invited me, maybe I was invited by your sister? He said ‘I don’t care, you are not going.’

### ‘The silent treatment:’ Poor or lack of communication

In both Rwanda and South Africa, women also described male partners deliberately not communicating with their spouses/girlfriends or preventing their partners from communicating with them. This played out differently in the two contexts. In Rwanda, several women described how their spouses forbade them to speak to them. This was related to the salient social norm that men were the heads of households and were able to disregard women’s decisions or input. As Olivier, a male partner of a couple, said at endline when reflecting on his changed behavior: “I thought that she should never question my actions and that she had nothing good to tell me. I thought that her ideas were useless and that I reserved the right to give orders at home.” A few participants in Rwanda identified such silencing as a form of violence, including a local leader at baseline: “If a woman is not allowed to speak because she is a woman that is gender based violence.” There was ample discussion of the common expectation for Rwandan women to remain quiet and humble, which could justify this ‘silent treatment’, and undermine resolution of partner conflicts. One female partner of a couple in Rwanda at baseline lamented how this ‘silent’ treatment, whereby her partner refused to communicate with her, limited her decision-making power in the household:

Mariette: But when I tell him ‘Why don’t you tell me, let us do such and such things, or let us renovate this wall of the house, rather than it is only me who think about all this?’ he keeps quiet and doesn’t answer me. When he doesn’t answer me, I cannot do anything.

In South Africa, a number of women described how their boyfriends went silent and stopped talking to them when they had arguments, which could cause emotional pain. For Thembeka, after arguing with her boyfriend about whether she could go out one evening, he refused to talk to her for over a week:

From that day onwards he stopped talking to me…I would return from work and cook. He would eat the food but he wouldn’t talk to me. The entire week went by still he wouldn’t talk to me, a new week started still he wasn’t talking to me.

Sthelo described something similar: “sometimes he wouldn’t talk to me, I would be at his home and he wouldn’t talk to me the entire week.” Alongside not talking for extended periods of time, participants in both South Africa and Rwanda described men regularly leaving their partners without telling them where they were going or when they would return:

Olwethu: Vusi [boyfriend] has moods …Sometimes he won’t tell me where he is going and then only tell me over the phone, or WhatsApp. Sometimes he just walks out of the house and not say where he is going.

(South Africa)

Seraphin (male partner of couple, endline): I woke up in the morning and left and didn’t consider telling her where I was going, and I knew I would come back home late in the evening. But I realized it is not a behaviour that can strengthen a household.

(Rwanda)

### Access to housing

In both settings, women described men’s control over housing as upsetting and causing them emotional pain. Yet this played out differently, given the divergent social contexts of the relationships in South Africa and Rwanda. In Rwanda, men limited their spouses’ access to their joint homes, underpinned by the strong social norm that women marry into a man’s home, and that men are the heads of households. Several women reported being kicked out of their homes by their husbands. Therese, a female partner of a couple in Rwanda, said at endline: “Also, we once again argued about something and then he told me ‘Get out of my house’ and then I went out and spent the night at his parents’ home.” This indicates an extreme form of control and power among men, despite the 1999 Rwandan law that protects married men and women’s equal rights to household property. One opinion leader at baseline identified a common practice of Rwandan women being expelled from their homes by their husbands, as a severe form of emotional abuse:

Evode: We received cases of women who spend nights outside of a locked door, isn’t that a kind of violence? You expel her out of the house, and therefore she goes to seek refuge somewhere else. She can even spend two or three nights there!

This form of abuse could include women being excluded from entering the home, related to jealousy or as a form of control. As Valentine, a women’s safe space facilitator noted:

The other one was saying: ‘because I am the one who can sell something, let me go to the market’ for example she would go to sell passion fruits. Now imagine at what time she would come from the market! So when she was back from there in the evening she would find that the old man is furious. He tells her: ‘you will not enter this house! You have spent a day away and you were with your other men!’

Some women also reported threats from their husbands of being kicked out of their homes. Such threats were raised as a particular form of stress and abuse for informally married women, as their rights to property or custody in the case of separation or divorce was not guaranteed. A women’s safe space facilitator commented:

Nzovu: Because they are not formally married, there is that insecurity. If he can buy a plot without calling her to sign for ownership, this means even if he dies today anyone can come and claim it because I have no evidence.

In South Africa the majority of young women did not live with their boyfriends, so could not be kicked out of joint homes. In contrast, some women described being locked in their boyfriend’s house when they went to visit them. The reasons women described that this happened were numerous, but often tied to men’s attempts to control women’s mobility and decision-making. Thobile had wanted to leave her boyfriend’s house, but he did not want this and locked her in when he left:

There is no going back because one day he took the key and locked the door. I had said I was leaving, he took the key and locked me in and he said he wanted to see how I was going to leave because he locked and left. It wasn't that he locked and stayed inside.

Similarly, Langa described wanting to leave her boyfriend’s shack to go home, but he locked her in to stop her leaving. She also described not trying to break out, because she feared the public shame and humiliation of being seen doing this:

Langa: …He locked the door. 1, 2, 3, 4 O’clock he wasn’t back, I tried phoning but he left his phone underneath the pillow; 5, 6 O’clock he wasn’t back …He came back at 8 O’clock …

Sthelo: couldn’t you escape through the window?

Langa: I thought about screaming but then again people would ask what was I doing there in the first place? …I just felt like crying.

In one instance Thembeka, who had her rent paid by her boyfriend, described being moved without notice by her partner from her shack to a different informal settlement, quite a long distance away. She had no say in the decision and would not have chosen to move. Thembeka’s boyfriend did this when he discovered that she was having an affair with someone at her work, and he believed he could stop the affair by moving her away.

## Discussion

This cross-comparative analysis illustrates that across two very different contexts with divergent populations and relationships, the categories of emotional IPV experienced by women were very similar, with comparable underlying drivers and intentions. The specific manifestations within each category of emotional IPV, however, were linked to differences in the social context of relationships and dominant social norms, nonetheless underpinned by similar patterns of gender inequalities, with men asserting control and power over women including women’s sexuality. Much of the emotional abuse in South African informal settlements was unpinned by the overall instability of the setting and relationships, which is not surprising given that cohabitation was rare and concurrent relationships with other partners were common. In Rwanda, in contrast, the majority of participants were married or co-habiting, in relatively stable, rural environments. Harmful gender norms for married or co-habiting couples, including a strong belief that a man is the unquestioned head of the household, underpinned much of the emotional IPV in this setting. These contextual differences explained how similar experiences—control, humiliation, silencing, access to housing—manifested somewhat differently in each setting.

Despite widespread reports of experiences of humiliation in Rwanda and South Africa, the most significant issue in both contexts that women described as abusive was to be *publicly* embarrassed and shamed, in ways that sought to devalue the primacy of one’s relationship as a wife or spouse (in Rwanda), and position of primary girlfriend (in South Africa). The *public* nature of humiliation relates to the common social norm that relationship issues and domestic disputes should be kept hidden, and the intention to de-value a spouse by transgressing this norm. In another analysis of the South African interviews [15], it was found intimate relationships that are publicly acknowledged to be a source of ‘respect and dignity’ for women, so when their partners publicly humiliated them, this undermined their accrued social status from being in the relationship. The described forms of public humiliation differed in the two contexts, with women in South Africa describing public humiliation most commonly when men publicly cheated and fought with them. In Rwanda, women emphasised being verbally humiliated and de-valued in comparison to other women and wives in particular in public. In both settings, men used language and actions as a way to de-value women’s role and position as either a primary partner or wife, with the intention of deliberately hurting and shaming.

In both South Africa and Rwanda, men’s attempts to limit women’s movement and ability to socialise were driven by similar patterns of men seeking to assert control over women’s autonomy, and by men’s jealousy and perceived fears of their spouses’/girlfriends’ infidelity. The precise ways men sought to limit women’s mobility varied according to the different social norms around women’s mobility and work. For instance, in South African informal settlements where women’s work was common, some men policed women’s travel to and from work through continual checking up, whereas in Rwanda excessive monitoring focused on access to markets and government meetings. For young women in South Africa, their ability to go out in the evenings, with friends, was often curtailed by partners, and the use of mobile phones was a central point in how men sought to establish control. In Rwanda, women returning home with items and money was an area of concern, given the expectation of men as providers and the overall devaluing of women’s ability to make economic contributions.

In South Africa and Rwanda, women reported their spouse/boyfriend blocking communication between one another. In Rwanda, women could be prohibited from speaking, which may be related to social norms which dictate that men should speak first when present with women, or that women should remain quiet, especially with men present [29]. In South Africa, it was more common for men to stop talking to women, rather than preventing women from speaking. In both cases, men asserted their power to stop discussion and communication in the relationship. Poor communication contributes to violence among intimate partners [30], and for women, communication can be a strategy to offset power imbalances during times of conflict [30]. We recognize the difference between these two forms of blocking communication; silencing is an exertion of power and control, whereas the silent treatment can be more nuanced with varying intentions. For instance, men’s silences can be strategic ways of limiting conflict emerging in relationships. Yet, negative consequences and interpretations of both forms of silencing were identified by women. When men shut down communication through the ‘silent treatment’ they curtailed women’s access to communication as a relationship strategy. While different methods of stopping communication were used in South Africa and Rwanda, related to different social contexts and dominant ideas about communication, the use of silencing by men in both settings were perceived as causing women emotional pain. The joint data highlights the role of control over communication, or specifically men’s foreclosing communication in a variety of ways, as neglected aspects of emotional IPV research. Typically, quantitative studies have focused more broadly on conflict or arguments as a context for emotional pain, whereas our analysis highlights the ways in which *silencing* can also be a form of emotional IPV.

Control over housing was another strategy that men used to control women’s mobility and associations in both settings, as well as inflict emotional pain. The patterning of how men used housing to do so was related to the different housing situations in each context. In South African women reported being locked into their boyfriends’ homes, as young women and men rarely cohabited, whereas in Rwanda, women reported being locked out or evicted from their joint home with their spouse. It is important to note however that other studies in other South African settings do provide evidence of women being evicted from their homes by their intimate partners as a form of abuse. For instance, a 3-province study found that 5.3% of women in the Eastern Province, 9.0% in Mpumalanga, and 3.6% in Northern Province reported being evicted from their homes by their intimate partner in the last year [31]. In this study, eviction from homes in Rwanda appeared to be related to the strong social norms that men are heads of households [20], which was codified by the law until 2016. In both settings, the use of housing (eviction or being locked in) represent severe expressions of men’s dominance and control. Housing is often not explicitly considered in qualitative research as a form of emotional IPV or is subsumed under overall control of women’s movements and should be given more explicit attention.

Overall, emotional IPV was common in both settings, with similar consequences. Consequences included undermining the self-worth of the abused partner, causing emotional harm, and impairing relationship quality. Relationship quality is often viewed as a composite of constructs such as relationship satisfaction, commitment, trust, intimacy, love and mutually constructive communication [32] and regarded as foundational for healthy behaviours within couples [29, 33]. Yet, despite the significant discussion of acts that were perceived as emotionally violent and identification of their serious consequences, it seems to have been a relatively normalized form of IPV in both study settings. The data suggested women had limited ability to report or seek help or support for emotional IPV.

This analysis illustrates the fluid and complex boundaries between emotional IPV, jealousy, controlling behaviours, and economic IPV. Quantitative studies sometimes make distinctions between emotional IPV and controlling behaviours [2]. Yet as shown in the qualitative data, there was no simple distinction between these different domains, which all had significant emotional impacts on women. Indeed, our data speaks to the value of and need for more qualitative, comparative studies on forms and consequences of emotional IPV. This could also helpfully inform surveys, as there is currently a lack of consensus for survey measures around emotional IPV and controlling behaviours [4]. Such research will also make important contributions to the field of IPV prevention and response as there is no unified agreement in the field on what constitutes emotional abuse [4]. Much more attention should also be given to the linkages between emotional and economic IPV.

### Strengths and limitations

As with all secondary analyses, the analysis presented here has limitations. Emotional IPV was not the sole focus of the topic guides in either settings, and the comparison between them was conducted as a *post hoc* analysis of sets of interviews that were designed to inform their respective impact evaluations and not for direct comparison. The lack of data from men in South Africa limited our ability to compare men’s perceptions of emotional IPV, including their experiences of emotional IPV, although the later was beyond the scope of this paper. The within-country social contexts differed between the settings, with South Africa interviewees being young adult in urban informal settlements, while the Rwandan participants had more age diversity and were largely drawn from rural villages. It is possible that some of the differences in specific manifestations of emotional abuse highlighted here stem from these differences in setting type rather than national differences, but this does not undercut our central argument about shared categories with difference in specific manifestations and may indeed affirm the need for finer-grained context specific information about emotional abuse to inform research and intervention. The analysis identified thematically grounded codes and categories to emerge for comparison, which we as authors categorized as emotional IPV, rather than relying on participant’s categorizations of emotional IPV. This may be a potential limitation but is also related to the challenge of people understanding and identifying emotional IPV. Another limitation of this study is not including data from men, which would be helpful to assess their own experiences of emotional IPV, as well as their motives for the forms of emotional IPV identified. This would be helpful to unpack intentions behind the differences in thematically identified domains of emotional IPV such as between silencing of women and the silent treatment, and between evicting to locking someone in their home.

As with all qualitative research, there was no attempt in either setting to generalize to the wider community or country from which the data are drawn. However, in alignment with qualitative best practices, participants in both settings were purposively sampled to ensure breadth of perspective, and in both cases data saturation was obtained with respect to emerging themes. Because the interviews were conducted at multiple time points while the interventions were taking place, the interventions may have influenced participants’ later conceptions and experiences of emotional IPV in Rwanda. Nonetheless, the data offer valuable insights into women’s categorial understanding of emotional abuse in the two contexts and this has enabled us to make visible many similarities, as well as areas of difference. The description of the contexts, analysis of detailed quotations, and the triangulation of the two diverse case studies also strengthened this secondary analysis. In addition, the authors aimed to be reflexive of their positionality to the data and understanding of the contexts. This was done through continual questioning of the data, and ongoing discussions with project team members in both contexts.

## Conclusion

The extent to which behaviors that constitute emotional IPV look different, and are embedded in different relationship dynamics, motives and consequences, can have important implications for how women experience IPV in different contexts and the validity of monitoring and evaluation efforts. Emotional IPV experienced by married or co-habitating women in Rwanda and young unmarried women in urban informal settlements in South Africa were surprisingly similar despite the differences in context and dominant forms of sexual relationships. Women in both contexts described public humiliation, control of mobility, access to housing and silencing as common experiences, which caused them emotional pain, which we can interpret as emotional IPV. Emotional IPV was regularly used to de-value women and assert men’s power, which indicates the importance of a gendered analysis of power regarding emotional IPV. Emotional IPV was also identified as extremely common in both settings, with considerable barriers to seeking help or even identifying it as violence. This is worrying given the significant health impacts of emotional IPV [4]. Overall, emotional IPV warrants more serious attention in violence prevention research and programming and should be given a much more prominent place in public discussion of partner violence. Interventions should be evaluated for how they prevent and respond to emotional IPV in addition to physical and sexual IPV. There is also a need to further understand how emotional IPV acts in synergy with physical and sexual violence to worsen health consequences. To inform such programming and evaluations, there is a particular case to be made for qualitative research around experiences and emic conceptualizations of emotional IPV within different contexts, and among different types of intimate relationships. Cross comparative studies including this one, indicate the value of such grounded, contextual analysis.

## Supporting information

S1 File Further details of primary research methods South Africa. (DOCX)

S2 File Further details of primary research methods Rwanda. (DOCX)

## References

[1] Follingstad DR. The Impact of Psychological Aggression on Women's Mental Health and Behavior: The Status of the Field. Trauma, Violence, & Abuse. 2009;10(3): 271–89. 10.1177/1524838009334453 19460760

[2] Durevall D, Lindskog A. Intimate partner violence and HIV in ten sub-Saharan African countries: what do the Demographic and Health Surveys tell us? The Lancet Global Health. 2015; 3(1):e34–e43. 10.1016/S2214-109X(14)70343-2 25539967

[3] Fulu E, Warner X, Miedema S, Jewkes R, Roselli T. and Lang J. Why Do Some Men Use Violence Against Women and How Can We Prevent It? Quantitative Findings from the United Nations Multi-country Study on Men and Violence in Asia and the Pacific. 2013, Bangkok: UNDP, UNFPA, UN Women and UN.

[4] Gibbs A, Dunkle K, Jewkes R. Emotional and economic intimate partner violence as key drivers of depression and suicidal ideation: A cross-sectional study among young women in informal settlements in South Africa. PLOS ONE. 2018;13(4):e0194885 10.1371/journal.pone.0194885 29659595 PMC5901771

[5] Jewkes R. Intimate partner violence as a risk factor for mental ill-health in South Africa Garcia-Moreno C, Riecher-Rossler A. Violence against Women and Mental Health, 65–74. Karger, 2013.

[6] Stockl H, Penhale B. Intimate Partner Violence and Its Association with Physical and Mental Health Symptoms Among Older Women in Germany. J Interpers Violence. 2015;30(17):3089–111. 10.1177/0886260514554427 25392386

[7] Jewkes R. Emotional abuse: a neglected dimension of partner violence. Lancet (London, England). 2010;376(9744):851–2. 10.1016/S0140-6736(10)61079-3 20822808

[8] World Health Organization. Multi-country study on women’s health and domestic violence: Initial results on prevalence, health outcomes and women’s responses. Geneva: World Health Organization, 2005.

[9] Gibbs A, Mushinga M, Crone E.T, Willan S, Mannell J. How do National Strategic Plans for HIV and AIDS in southern and eastern Africa integrate gender-based violence? Health and Human Rights. 2012, 14(2): 1–11. 23568943

[10] Gibbs A, Jewkes R, Willan S, Washington L. Associations between poverty and intimate partner violence amongst young women and men in urban informal settlements in South Africa: a cross-sectional study and structural equation model. PLoS One. 2018; 13(10): e0204956. 30281677 10.1371/journal.pone.0204956 PMC6169941

[11] Machisa M, Jewkes R, Morna C, Rama K. The war at home: Gender based violence indicators project. Gauteng Research Report. Johannesburg, South Africa: Gender Links & South African Medical Research Council, 2011.

[12] Hunter M. The changing political economy of sex in South Africa: the significance of unemployment and inequalities to the scale of the AIDS pandemic. Social Science & Medicine. 2007; 64(3):689–700. 17097204 10.1016/j.socscimed.2006.09.015

[13] HDA. KwaZulu-Natal: Informal settlements status. Johannesburg: HDA, 2012.

[14] Gibbs A, Washington L, Willan S, Ntini N, Khumalo T, Mbatha N, et al The Stepping Stones and Creating Futures intervention to prevent intimate partner violence and HIV-risk behaviours in Durban, South Africa: study protocol for a cluster randomized control trial, and baseline characteristics. BMC Public Health. 2017;17(1):336 10.1186/s12889-017-4223-x 28427380 PMC5397780

[15] Willan S, Ntini N, Gibbs A, Jewkes, R. “If he loves you he can hit you, but not injure you.” Exploring young women’s constructions of love and strategies to navigate violent relationships in South African informal settlements. Culture, Health & Sexuality. 2019. 10.1080/13691058.2018.1554189 30632915

[16] Umubyeyi A, Mogren I, Ntaganira J, Krantz G. Women are considerably more exposed to intimate partner violence than men in Rwanda: results from a population-based, cross-sectional study. BMC Women's Health. 2014;14:99 10.1186/1472-6874-14-99 25155576 PMC4148406

[17] Slegh H, Kimonyo, A. Masculinity and Gender Based Violence in Rwanda: Experiences and Perceptions of Men and Women. Kigali: RWAMREC, 2010.

[18] Umubyeyi A, Persson M, Mogren I, Krantz G. Gender Inequality Prevents Abused Women from Seeking Care Despite Protection Given in Gender-Based Violence Legislation: A Qualitative Study from Rwanda. PLOS ONE. 2016;11(5): e0154540 10.1371/journal.pone.0154540 27152680 PMC4859471

[19] National Institute of Statistics Rwanda. Rwanda Demographic and Health Survey. Kigali, Rwanda: Ministry of Finance and Economic Planning, 2014–2015.

[20] Sommers M. Stuck: Rwandan Youth and the Struggle for Adulthood. Athens, GA: University of Georgia Press, 2012.

[21] Powley E. Rwanda: The impact of women legislators on policy outcomes affecting children and families. State of the World’s Children Background Paper, UNICEF, 2007.

[22] Polavarapu A. Procuring Meaningful Land Rights for the Women of Rwanda. Yale Human Rights and Development Law Journal. 2011; 14(1): 3.

[23] Kaiser Hughes A, Ndangiza M, Ikirezi M. The rights of women in de facto unions to land and property. Kigali, Rwanda: USAID Land Project. 2016.

[24] Uwineza P, Pearson E. Sustaining Women’s Gains in Rwanda: The Influence of indigenous Culture and Post-Genocide Politics. Washington, DC: Institute for Inclusive Security, 2009.

[25] Stern E, Niyaratunga, R. A process review of the Indashyikirwa couples curriculum to prevent intimate partner violence and support healthy, equitable relationships in Rwanda. Journal of Social Sciences: Special Edition on Select Papers from Conference on Global Status of Women and Girls. 2017; 6(63).

[26] Stern E, Martins S, Stefanik L, Uwimphuwe S, Yaker R. Lessons learned from implementing Indashyikirwa in Rwanda- an adaptation of the SASA! approach to prevent and respond to intimate partner violence. Evaluation & Program Planning. 2018, 71: 58–67. 30125773 10.1016/j.evalprogplan.2018.08.005

[27] Braun V, Clarke V. Using thematic analysis in psychology. Qualitative Research in Psychology. 2006;3(2):77–101.

[28] Bartlett L, Vavrus F. Comparative case studies: An innovative approach. Nordic Journal of Comparative and International Education (NJCIE). 2017; 1(1): 5–17.

[29] Carlson K, Randell S. Gender and development: Working with men for gender equality in Rwanda. Agenda. 2013;27(1):114–25.

[30] Conroy AA, McGrath N, van Rooyen H, Hosegood V, Johnson MO, Fritz K, et al Power and the association with relationship quality in South African couples: Implications for HIV/AIDS interventions. Social Science & Medicine. 2016;153:1–11. 26859436 10.1016/j.socscimed.2016.01.035 PMC4788545

[31] Jewkes R, Penn-Kekana L, Levin J, Ratsaka M, Schrieber M. Prevalence of emotional, physical and sexual abuse of women in three South African provinces. SAMJ. 2001, 91(5): 421–428. 11455808

[32] Fletcher GJO, Simpson JA, Thomas G. The Measurement of Perceived Relationship Quality Components: A Confirmatory Factor Analytic Approach. Personality and Social Psychology Bulletin. 2000; 26(3):340–54.

[33] Lewis MA, McBride CM, Pollak KI, Puleo E, Butterfield RM, Emmons KM. Understanding health behavior change among couples: an interdependence and communal coping approach. Social Science & Medicine. 2006; 62(6):1369–80. 16146666 10.1016/j.socscimed.2005.08.006

