# Supplementary material for: ‘When you talk to someone in a bad way or always put her under pressure, it is actually worse than beating her’: Conceptions and experiences of emotional intimate partner violence in Rwanda and South Africa
Source: PLoS One. 2019 Nov 14;14(11):e0225121. doi: 10.1371/journal.pone.0225121 (PMC6855458; doi:10.1371/journal.pone.0225121)
Supplement: S1 File — (DOCX) [file pone.0225121.s001.docx]

***‘When you talk to someone in a bad way or always put her under pressure, it is actually worse than beating her’*: Conceptions and experiences of emotional intimate partner violence in Rwanda and South Africa**

**S1 File. Further Details of Primary Research Methods South Africa**

Data were collected between May 2016 and February 2017 from a group of women from two communities participating in a randomised control trial (RCT) of a Stepping Stones, Creating Futures intervention that aimed to reduce IPV and HIV-risk [1]. Qualitative methods were utilised for their value in meaningfully involving marginalised groups, and their ability to provide insight into women’s social world, offering an in-depth understanding of social context, perspectives and experiences [2, 3].

**Setting**

The study took place in two urban informal settlements (slums) in eThekwini Municipality. The two communities from which women were drawn were broadly similar, allowing for comparability across themes. Both were very poor, with high rates of unemployment – a reported 39% of young people were unemployed in 2011 in the eThekwini Municipality and low levels of education, overcrowding and limited or no services or formal housing. Despite being similar, the communities differed slightly from one another: the first had slightly better housing and taps in the yards. The second community had more dilapidated housing and shacks, and only communal taps.

**Methods**

Semi-structured, in-depth interviews (IDIs) were undertaken with 15 women. The interviews were supplemented by photovoice methodology1 in one community (involving eight of the women) and light-touch participant observation in the other community (seven of the women).

**Participants**

The women were recruited through convenience sampling. Eligibility for participation in the study included being unemployed, aged between 18–30 and not being in school or college (linked to the livelihoods focus of the larger RCT intervention). Interviews were conducted before or just as the intervention that took place as part of the larger RCT study began. Just over half had children, none were in formal employment and only two had completed secondary school. Women were asked about their relationships in open-ended questions, in isiZulu, and most stated that they were in love-relationships at the time of being interviewed. Three were living with their partners, two others moved between their natal home and their partner’s and one was married. To ensure confidentiality we use pseudonyms throughout this paper, and identifying information has been removed.

**Data collection**

Each woman participated in two interviews (a total of 30 interviews). The first involved open-ended questions about her life focused on building trust, and the second, usually conducted within two weeks, focused on relationships, power and decision-making. Interview topics included: background information on their lives, relationships, experiences of IPV, livelihoods, power and aspirations. Each interview lasted between 45 and 90 minutes.

All 15 women participated in either the photovoice or the participant observation process. Data collected through these different forms of observation were used to enhance our understanding of the community dynamics and the women’s lives. The interviewer (NN) kept notes of her observations in the field. For women involved in the photoshop element of the study, three 3-hour workshops were held, with two periods in between for taking photos. In all, the women produced 24 photo-posters of their lives. All the interviews and photovoice sessions were conducted by the second author in isiZulu, and translated and transcribed into English. The research was guided by the WHO (2001) guidance for research in this area (5). In cases where participants became distressed or reported being emotionally affected by the research, they were offered access to a counsellor and/or referred to an appropriate service provider. Among the women involved in the qualitative research, one woman was referred to a service provider when she requested additional support following a discussion with the research team; she reported that the process of discussion helped her to reflect on her situation, and she wished to seek further counselling.

**References**

1. Willan S, Ntini N, Gibbs A, Jewkes, R. “If he loves you he can hit you, but not injure you.” Exploring young women’s constructions of love and strategies to navigate violent relationships in South African informal settlements. Culture, Health & Sexuality. 2019.
2. Dicicco-Bloom, B., and B. F. Crabtree. 2006. “The Qualitative Research Interview.” Medical Education 40 (4): 314–21. doi:10.1111/j.1365-2929.2006.02418.x
3. Sprague, C. 2018. Gender and HIV in South Africa: Advancing Women’s Health and Capabilities. Basingstoke: Palgrave Macmillan.
4. World Health Organisation 2001. “Putting Women First: Ethical and Safety Recommendations for Research on Domestic Violence against Women.” http://www.who.int/gender-equity-rights/ knowledge/who_fch_gwh_01.1/en/
