# Supplementary material for: ‘When you talk to someone in a bad way or always put her under pressure, it is actually worse than beating her’: Conceptions and experiences of emotional intimate partner violence in Rwanda and South Africa
Source: PLoS One. 2019 Nov 14;14(11):e0225121. doi: 10.1371/journal.pone.0225121 (PMC6855458; doi:10.1371/journal.pone.0225121)
Supplement: S2 File — (DOCX) [file pone.0225121.s002.docx]

***‘When you talk to someone in a bad way or always put her under pressure, it is actually worse than beating her’*: Conceptions and experiences of emotional intimate partner violence in Rwanda and South Africa**

**S2 File. Further Details of Primary Research Methods Rwanda**

This paper presents ongoing feedback gathered from Indashyikirwa participants and from RWN and RWAMREC program staff, which was conducted as part of the impact evaluation of the program [1]. The interviews with participants took place in three intervention sectors (Rurembo Sector, Western Province; Gishari Sector, Eastern Province; and Gacaca Sector, Northern Province), which were purposefully selected to represent a diversity of environments including rural and peri urban locations. As self-report data may be limited by the ability of participants to accurately recall information [2] participants were interviewed at different intervals throughout the program. In November 2015, thirty interviews were conducted separately with both partners of couples enrolled in but before having begun the Couples Curriculum. The first author informed staff from the Rwandan research company Laterite, which conducted the randomized control trial with couples, of recruitment criteria, and aims of the study, so that these could be disseminated to potential participants. Laterite staff provided the qualitative researchers with couples’ contact details after obtaining their consent to do so. Couples were purposefully selected to include a diversity of informally and formally married couples, for being the primary distinction among couples enrolled in the curriculum. The interviews assessed couples’ expectations of the program, their experiences of conflict and IPV, communication skills and joint decision-making. Twenty-eight midline interviews were conducted with the same sub-set of couples immediately after the curriculum in May 2016 (due to one couple being lost to follow up) to assess their impressions of and impact of the curriculum. Twenty-eight endline interviews were also conducted with the same sub-set of couples in May 2017, one year after the midline interviews. Couples were asked how their involvement with Indashyikirwa has continued to impact their relationships.

Nine baseline interviews (three per sector) were conducted with opinion leaders enrolled in and before completing the Indashyikirwa opinion leader module in November 2015. RWN staff members purposefully suggested a diversity of opinion leaders to include government leaders, members of anti-GBV committees or the National Women’s Council and religious leaders. Opinion leaders were asked about their expectations of the Indashyikirwa program, and their experiences around IPV prevention and response. Six midline interviews were conducted with the same sub-set of opinion leaders after twelve months (November 2016), as three opinion leaders were lost to follow up due to being replaced as local leaders after re-elections. Three additional opinion leaders were interviewed in June 2017, after completing a refresher training and being incorporated into the program. These interviews assessed opinion leaders’ impressions of the Indashyikirwa training and whether their involvement in the program has influenced their actions for IPV prevention and response.

In May 2016, three women’s safe space facilitators (WSF) (one per safe space) were interviewed to assess their motivations as facilitators and their impressions of the training they received. RWN staff supported recruitment of WSF and attendees. In September 2016, six women who attend the safe spaces (two per sector) were interviewed to assess why they visit the safe spaces, and the difference the spaces make in their lives (if any). In June 2017, three different WSF (one per sector) were interviewed to assess their perceived impact of the safe spaces and the support they receive as facilitators. Six female attendees (two per sector), one male attendee in the Northern Province and one male attendee in the Western Province were also interviewed to assess their impressions of the women’s safe spaces. Twelve partners of couples who were elected and trained as CAs (four per sector) were interviewed in November 2016, after having completed the activist training and started conducting activism activities. They were recruited through RWAMREC staff and were asked about their impressions of the activism training, what motivated them to continue as CAs, what they had been doing recently as CAs, and whether they had faced any challenges.

In May 2016, six in-depth interviews were conducted with RWN field officers and supervisors across all intervention sectors, which assessed their perspectives of successes and lessons learned from facilitating the opinion leader and women’s space facilitator modules. Another round of interviews were conducted with seven RWN staff in May 2017, where they were asked to describe key successes and challenges of the women’s safe spaces and engagement of opinion leaders. Interviews were conducted with ten RWAMREC field officers and supervisors in May-June 2016 across all intervention sectors, which assessed their perspectives of successes and lessons learned from facilitating the Couples Curriculum. Eight RWAMREC field supervisors and officers were also interviewed in May 2017, where they were asked to describe key successes and challenges of the community activism component. The interviews with program beneficiaries and staff lasted approximately 1–1.5 hours and were conducted at locations deemed appropriate and private for participants. Two female Rwandan qualitative researchers external to the program conducted the interviews with WSFs, attendees, opinion leaders, CAs and female partners of couples. Two male Rwandan qualitative researchers conducted the interviews with opinion leaders and male partners of couples. All of these interviews were conducted in Kinyarwanda and audio recorded. The first author conducted both rounds of interviews with RWAMREC and RWN staff in English.

**References**

1. Stern E, Martins S, Stefanik L, Uwimphuwe S, Yaker, R. Lessons learned from implementing Indashyikirwa in Rwanda- an adaptation of the SASA! approach to prevent and respond to intimate partner violence. Evaluation & Program Planning. 2018, 71: 58-67.
2. James Bell Associates. Evaluation brief measuring implementation fidelity. 2009, Arlington Virginia.
